# Inflammatory myositis in the pediatric rheumatology clinical practice – a case series

**DOI:** 10.1186/1546-0096-9-S1-P59

**Published:** 2011-09-14

**Authors:** S Melo Gomes, M Conde, MP Ramos, JA Melo Gomes

**Affiliations:** 1 Department of Pediatrics, Centro Hospitalar Oeste Norte, Portugal; 2 Department of Pediatrics,Hospital de Dona Estefânia, Portugal; 3 Pediatric Rheumatology Clinic, Instituto Português de Reumatologia, Lisbon, Portugal

## Background

Inflammatory myopathies (IM) in children comprise a heterogeneous group of disorders, the most common being juvenile dermatomyositis and to a lesser degree juvenile polymyositis.

## Aim

To assess the clinical characteristics and treatment response of a cohort of IM patients.

## Methods

Clinical chart review of clinical, laboratory and treatment related parameters of IM patients treated at 2 referral centers for the last 12 years.

Outcome measures included disease remission and muscular function.

## Results

17 IM patients (12F/5M, median age at diagnosis-8years(2-16years)) were followed for a mean of 6,3years (1-12years): 3 were labeled as polymyositis and 14 as juvenile dermatomyositis.

Positive diagnostic criteria: typical skin lesions-14/17, proximal muscle weakness-15/17, elevated muscle enzymes-16/17, EMG-10/10, muscle biopsy-9/9.

Frequent presenting symptoms included: proximal muscle weakness-16/17, skin lesions-11/17, lethargy-8/17, fever-7/17. During follow-up, patients presented with: muscle weakness (17/17), skin lesions (14/17), lipodystrophy (2/17), arthralgia (6/17), arthritis(4/17), vasculitis (4/17), gastro-intestinal vasculitis (1/17), restrictive pulmonary disease (1/17), calcinosis (4/17).

Laboratory: ESR was raised in 6/17, muscle enzymes in 17/17 (CK-16/17, median-1149; LDH 16/17, median-875; aldolase-7/17); Positive auto-antibodies: ANA-11/17, SSA(Ro-52)-2/17; 10/17 underwent muscle biopsy and 9/17 EMG.

Therapeutic regimens included more commonly steroids, methotrexate (17/17) and CyclosporinA (16/17).

Regarding disease activity, 9/17 patients have inactive disease, 6 of which are in remission without treatment; 6/17 have permanent loss of muscular function.

## Conclusion

IM are potentially severe, incapacitating diseases. All patients with polymyositis in this series have loss of muscular function, contrasting with 3/14 of JDM patients.

About half of this cohort is asymptomatic and it should be stressed that early diagnosis and aggressive treatment are important prognostic factors.

